# Design, Screening, and Testing of Non-Rational Peptide Libraries with Antimicrobial Activity: In Silico and Experimental Approaches

**DOI:** 10.3390/antibiotics9120854

**Published:** 2020-11-30

**Authors:** Paola Ruiz Puentes, María C. Henao, Carlos E. Torres, Saúl C. Gómez, Laura A. Gómez, Juan C. Burgos, Pablo Arbeláez, Johann F. Osma, Carolina Muñoz-Camargo, Luis H. Reyes, Juan C. Cruz

**Affiliations:** 1 Center for Research and Formation in Artificial Intelligence, Universidad de los Andes, Bogota DC 111711, Colombia; p.ruiz@uniandes.edu.co (P.R.P.); pa.arbelaez@uniandes.edu.co (P.A.); 2 Department of Biomedical Engineering, Universidad de los Andes, Bogota DC 111711, Colombia; ce.torres10@uniandes.edu.co (C.E.T.); sc.gomez11@uniandes.edu.co (S.C.G.); la.gomez14@uniandes.edu.co (L.A.G.); c.munoz2016@uniandes.edu.co (C.M.-C.); 3 Grupo de Diseño de Productos y Procesos, Department of Chemical and Food Engineering, Universidad de los Andes, Bogota DC 111711, Colombia; mc.henao10@uniandes.edu.co; 4 Chemical Engineering Program, Universidad de Cartagena, Cartagena 130015, Colombia; jburgosb@unicartagena.edu.co; 5 Department of Electrical and Electronic Engineering, Universidad de los Andes, Bogota DC 111711, Colombia; jf.osma43@uniandes.edu.co; 6 School of Chemical Engineering and Advanced Materials, The University of Adelaide, Adelaide 5005, Australia

**Keywords:** antimicrobial peptides, antibiotic resistance, deep learning, molecular dynamics, microfluidics, library screening, rational, non-rational

## Abstract

One of the challenges of modern biotechnology is to find new routes to mitigate the resistance to conventional antibiotics. Antimicrobial peptides (AMPs) are an alternative type of biomolecules, naturally present in a wide variety of organisms, with the capacity to overcome the current microorganism resistance threat. Here, we reviewed our recent efforts to develop a new library of non-rationally produced AMPs that relies on bacterial genome inherent diversity and compared it with rationally designed libraries. Our approach is based on a four-stage workflow process that incorporates the interplay of recent developments in four major emerging technologies: artificial intelligence, molecular dynamics, surface-display in microorganisms, and microfluidics. Implementing this framework is challenging because to obtain reliable results, the in silico algorithms to search for candidate AMPs need to overcome issues of the state-of-the-art approaches that limit the possibilities for multi-space data distribution analyses in extremely large databases. We expect to tackle this challenge by using a recently developed classification algorithm based on deep learning models that rely on convolutional layers and gated recurrent units. This will be complemented by carefully tailored molecular dynamics simulations to elucidate specific interactions with lipid bilayers. Candidate AMPs will be recombinantly-expressed on the surface of microorganisms for further screening via different droplet-based microfluidic-based strategies to identify AMPs with the desired lytic abilities. We believe that the proposed approach opens opportunities for searching and screening bioactive peptides for other applications.

## 1. Introduction

Antimicrobial resistance (AMR), both inherent and acquired, has become an issue of increasing concern in recent years. AMR negatively impacts population health and healthcare systems costs and gross domestic product (GDP) [1]. Inherent resistance is a natural attribute that protects the organism from antimicrobials (AM), such as the Gram-negative bacteria’s outer membrane. Contrarily, acquired resistance is caused by genetic mutations that enable the microorganism to resist antimicrobials through different underlying mechanisms. Within those mechanisms, some of the most important include drug inactivation by enzymes, cell wall modifications, alteration of AM targets’ binding sites, efflux pumps that expel the AM bypassing the targets, and modification of metabolic pathways [2]. AMR is the consequence of misuse and overuse of antibiotics, self-medication, self-interrupted treatments, exposure to nosocomial infections in hospitals, genetic plasticity, and sheer dogged adaptability of the microorganisms themselves [2,3]. To complicate the situation even further, the pharmaceutical industry has virtually stopped developing new antibiotics, mainly due to economic and regulatory obstacles. By 2015, 15 out of 18 of the largest pharmaceutical companies had abandoned the antibiotic field [4]. This lack of new molecules to treat infections has been related to a new pre-antibiotic era, in which infections and minor injuries which have been treatable for decades may once again kill millions [5]. Recent studies have estimated that by 2050, more than ten million deaths per year are attributed to resistant pathogens, with a higher percentage of them occurring in developing countries [6].

Specifically, for bacteria, by 2016, the World Health Organization (WHO) reported that the global incidence of infection cases approached 490,000, which can be attributed to these resistant pathogens [5]. In 2019, just in the USA, more than 2.8 million antibiotic-resistant infections were reported by the CDC (Center for Disease Control and Prevention), which resulted in more than 35,000 deaths [7]. These statistics clearly show the exponential increase in resistant bacteria. The ESKAPE group comprises six nosocomial multidrug-resistant microorganisms (*Enterococcus faecium*, *Staphylococcus aureus*, *Klebsiella pneumoniae*, *Acinetobacter baumannii*, *Pseudomonas aeruginosa*, and *Enterobacter* spp.) and it is listed by the WHO as a priority to acquired new antibiotics. Within this group, the most problematic ones are perhaps the carbapenem (last resort family of antibiotics) resistant *A. baumannii*, *P. aeruginosa*, *K. pneumoniae*, and *Enterobacter* spp. and for that reason, they are listed with critical priority. Additionally, concerning are the vancomycin-resistant *E. faecium* and methicillin and vancomycin-resistant *S. aureus*, which are listed with high priority. ESKAPE pathogens are responsible for most nosocomial infections and represent the vast majority of isolates whose resistance presents serious therapeutic dilemmas to physicians such as experimental treatment selection, comorbidities treatment, especially cancer, and isolation procedures [3,8]. Compared to non-ESKAPE pathogens, the ESKAPE group has shown a higher mortality rate and higher costs due to the need for more comprehensive and sophisticated treatments [9].

Furthermore, there are reports of resistance incidences against some of the more newly discovered/designed antibiotics, and the outlook appears not to improve in the coming years. Therefore, an imperative is to find alternative treatments, especially for the ESKAPE pathogens [3]. Moreover, there is a concern because these infections are no longer confined to hospitals. Over recent years, rising resistant infections in the community have been detected, which can put more people at risk, in addition to making the spread more challenging to identify and contain [10].

Virus resistance is a less concerning issue. However, some important or common viruses, such as influenza, hepatitis C, herpes, and human immunodeficiency virus (HIV), exhibit AM resistance. HIV has shown inherent resistance to some antiretrovirals (ARV) via some proteases and reverse transcriptases [11]. Some countries have recently reported AM levels at or above 15% among patients starting ARV treatment and 40% among patients after re-starting treatment. This shows that HIV has also acquired resistance, which has led to substantial economic implications, given that second and third-line treatments are three times and 18 times more expensive, respectively [5]. Influenza virus has a high mutation rate; therefore, it easily achieves resistance to most commonly used antivirals such as adamantanes and the neuraminidase inhibitors (NAIs). Furthermore, in 2018 resistance to favipiravir, a broad-spectrum antiviral, was reported in vitro, suggesting that a possible unreported resistance mechanism could exist in the worldwide population [12].

An alternative treatment for all the resistant microorganisms described above has emerged in the last decade: the antimicrobial peptides (AMPs). AMPs are short peptides with a broad spectrum of antimicrobial activities that are part of living organisms’ defense mechanisms against microbial pathogens. Since AMPs have diverse chemical features and cellular targets, they are promising AM agents with an expected lower rate of acquired resistance by microorganisms [13]. The action of most antimicrobial peptides relies on the interaction between the positive charges in the peptide’s residues and the negatively charged membrane components. The structural and physicochemical properties of antimicrobial peptides and their capacity to adopt an amphipathic conformation upon membrane binding influence this interaction [13]. This conformation results from a balance between positively charged and hydrophobic amino acid residues [14]. The insertion of antimicrobial peptides into the membrane’s hydrophobic core depends on the microbe membrane [13].

Regarding bacteria, the interaction with AMPs varies between Gram-negative and Gram-positive microorganisms. Cationic AMPs have shown to cross the outer membrane of Gram-negative bacteria by a charge-exchange mechanism of competition with membrane-bound Ca^2+^ and Mg^2+^. Upon interaction, the peptides bind to lipopolysaccharides, most likely promoted by the binding to outer membrane proteins, thereby reaching the cell membrane [15]. In contrast, given the cell wall’s porosity of Gram-positive bacteria, many AMPs seem to pass relatively easily. Once on the cell surface, single-cell studies have shown the accumulation of AMPs to be restricted to foci associated with cell division, cell wall remodeling, or secretion, thereby interfering with these vital processes or causing cell lysis [16]. Cationic AMPs amphipathic conformation allows increased interaction with the negatively charged surfaces or direct insertion into the bacterial membranes. Additionally, the higher potential inside the negative transmembrane in bacteria further enhances the strength of electrostatic attraction. The AMP–membrane interaction has been typically associated with barrel-stave, carpet, or toroidal-pore models [14].

Studies have shown that a single peptide can act through several mechanisms mediated by the topology, aggregation, and lipid interactions of AMPs with cellular membranes. These, in turn, rely on the peptide structure, the peptide/lipid ratio, and the properties of the lipid membrane [17]. Additionally, depending on AMPs concentration, the cellular membrane can rather expand, which results in pores that allow the transport of the peptide into the microorganism or generate local or massive ruptures of the membrane. Local perturbances induced by the peptides are due to interactions with proteins, nucleic acids, and cellular organelles, which by itself constitutes a potential cell-killing mechanism. The ability of individual AMPs to interact with multiple targets or multiple peptides to interact with a single target may limit the development of bacterial resistance [14].

Nowadays, peptides discovery is facilitated through library screening via both rational and non-rational approaches. There are three major methods for rational design: template-based design, physicochemical, and de novo methods, aiming to create novel peptides and/or improve existing ones. The template-based design aims to add selectivity and/or increase a known peptide sequence activity by including an amino acid or changing its position. This generally results in a reduction in the peptide sizes. With this approach, it is possible to identify novel AMP sequences even from inactive peptides. The physicochemical design also generates analogs with different physicochemical properties from known sequences. Finally, the de novo method creates new peptides based on amino acid patterns or frequencies [18].

The de novo method creates new peptides based on amino acid patterns or frequencies [18]. This approach is based on identifying sequence patterns, crucial residue positions, and amino acid frequencies from known AMPs. This information is then used to develop prediction methods and linguistic models to identify novel AMPs [19]. Generally, de novo AMP design involves favoring an amphipathic structure such that the peptide sequences exhibit both hydrophobic and hydrophilic regions [20]. Furthermore, de novo design can also be completed aided by machine learning methods such as variational autoencoders (VAEs) and generative adversarial networks (GANs). In the case of VAEs, the input data serves as the basis to create a continuous latent space that can be used to further interpolate between objects. As a result, by interpolating between two known AMPs it is possible to generate novel chemical structures that represent a smooth transition between both peptides. Dean and colleagues used this approach to obtain novel AMPs that where successfully validated experimentally [21]. In the case of GANs, the distribution of the input peptides is followed to generate a set of new ones through a two machine learning networks: the generator, that generates the new peptide sequences and the discriminator, whose task is to try to discriminate between real and fake peptides. In this way, at the end of the training the generated example peptides should not be discriminated as false and exhibit similar properties to the real ones [22].

In contrast, non-rational approaches rely on the microbial surface display technology for obtaining various well-established random peptide sequences for further screening according to the available methods within the framework of the peptide-based drug discovery process [23]. Combinatorial chemistry has been used to create libraries of peptides/proteins and discover new recombinant therapeutics [24]. Combinatorial library methods can be generated by vastly diverse chemical libraries, including phage display, yeast display, bacteria display, mRNA display, and more [25]. This technique’s primary strength is its capability to generate the enormously diverse exogenous peptides or proteins displayed on the cell’s surface using standard yet rapid molecular biology methods instead of using genetically engineered protein or peptide variants individually [26]. Guralp and colleagues proposed a five-step light-directed in situ parallel oligonucleotide synthesis with a cellular expression and screening system. The first step involves the AMP library design, where designed peptides and reverse-translated to oligonucleotides. In the second step, the library is synthesized by parallel synthesis technology which allows large numbers of oligonucleotides to be produced on a single array. In the third step, emulsion PCR is employed to amplify each of the oligonucleotides followed by their cloning and expression to find the bioactive peptide sequences. Finally, microorganism strains showing AMPs activities are chosen and their plasmids extracted for DNA sequencing and subsequent identification of the AMP candidates in silico [27]. Peptides have profoundly impacted the modern pharmaceutical industry’s development and have contributed significantly to biological and chemical science [24].

Furthermore, phage display technology, a combinatorial screening approach, provides a molecular diversity tool [24] for creating libraries of random peptides and proteins to identify ligands for receptors, identify enzyme blockers, studying protein/DNA–protein interactions, screening cDNA expression, epitope mapping of antibodies, engineering human antibodies, optimizing antibody specificities, identifying peptides that home to specific organs or tissues, generating immunogens for vaccine design, and use in affinity chromatography [26]. Phage display has several advantages over traditional random screening methods used in drug discovery, such as simplicity, cost-effectiveness, and speed [26].

The cell-surface display allows peptides to be displayed on microbial cells’ surface by fusing them with the anchoring motifs, usually cell-surface proteins or their fragments. The fusion can be accomplished by N-terminal fusion, C-terminal fusion, or sandwich fusion. The characteristics of carrier protein, passenger protein, and the host cell and fusion method might impact the efficiency of surface display of proteins [28].

**Figure 1:**
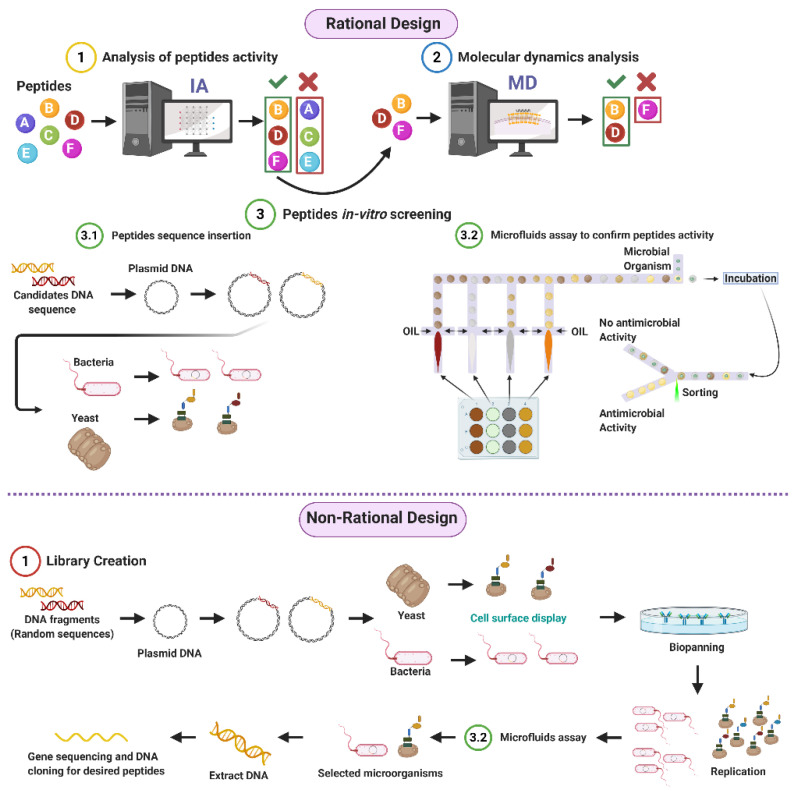
Antimicrobial Peptides (AMPs) discovery framework. Rational design steps: (I) Deep learning techniques identify sequences with potential antimicrobial activity, (II) membrane-disruption capabilities of selected sequences are analyzed via molecular dynamics (MD), (III) the host cell is modified, and sequences are inserted, finally (IV) antimicrobial activity is corroborated by a microfluidic system. Non-rational design steps: (I) Random sequences are expressed on host cells through cell surface display, (II) modified microorganisms are analyzed by a microfluidics system to obtain AMPs candidates, and (III) DNA is extracted, sequenced, and cloned (Created with BioRender).

Given the potential of AMPs to address the worldwide concern on resistant organisms, our research group proposes both rational and non-rational frameworks for a more efficient and faster method to find antimicrobial peptides. Our approaches rely on bacteria/yeasts surface display and low-cost microfluidics for screening such that experimentation costs are reduced without compromising throughput (Figure 1). Regarding rational design, the framework consists of a four-step process that aims to minimize time and resources, taking only the most promising peptides to experimental evaluation (Figure 1). The workflow begins with two computational phases: the first one comprises deep learning techniques to find sequences with potential antimicrobial activity. In the second one, the candidates are subjected to interaction with a cell membrane in silico via molecular dynamics (MD). This approach allows us to identify whether a candidate has the membrane-disruption capabilities required to be an AMP. Subsequently, the sequence is passed to the experimental phase where (I) the host with which the analysis will be carried out is modified by following the current molecular biology methods for surface display, and (II) a microfluidic system is used to corroborate the antimicrobial activity. Alternatively, for non-rational design, the framework consists of a three-step process: (I) Cell surface display, followed by (II) microfluidics analysis, and (III) DNA sequencing.

The first stage’s deep learning algorithm is based on recurrent neural networks (RNNs) composed of several layers, enabling learning data representations with multiple abstraction levels. The algorithm was inspired by natural language processing (NLP) techniques considering their suitability for problems based on the sequence’s involved elements. In this way, the generated representations can be easily interconverted into simpler ones. The reliability of these architectures has been previously demonstrated in property prediction and generation of molecules with certain features of interest [29,30,31,32]. The initial layers of the RNNs are capable of learning local information, while the deeper layers are focused more on learning global and abstract information [33]. For example, the initial layers will learn features representing functional groups or amino acids present in the peptides. In contrast, the deeper layers will learn features related to the amino acids’ sequence and the peptide’s global structure, which will enable predictions about their biological activity. The deeper layers take the initial layers’ as input information and combine them through mathematical operations to achieve that level of abstraction. Finally, for the learning process to be possible, a backpropagation algorithm is implemented to minimize an error function established at the beginning of the training process by adjusting each layer’s internal parameters iteratively [33].

Regarding the second stage, peptides–membrane interaction analysis computational simulations provide a powerful tool to understand different molecules’ properties through their interaction at a molecular and nanoscale [34]. These simulations provide missing information on the mechanistic details at the molecular scale of such interactions. Therefore, this approach closes a knowledge gap concerning the macroscopic information collected experimentally [35]. Moreover, it provides additional insights into controversial or counterintuitive results obtained at the macroscopic scale [36]. To achieve an understanding of the system at the atomic level, diverse techniques have been used, where Monte Carlo (MC) [37,38] and molecular dynamics (MD) rank high among the preferred choices [39,40]. These methodologies emerged in the late 1950s when Alder and Wainright published the first description of these tools, which were used to analyze the phase transition for hard-sphere systems [41]. Since then, they have evolved, becoming more accessible and powerful and reaching out to various research areas, including chemistry, materials science, biology, geology, and physics [42,43]. The main goals are to understand the interactions among several molecules involved in a particular situation and guide new experimental strategies toward a desired state by the insights provided by the simulations [44].

MC simulations have attracted significant attention for a deeper understanding of interactions due to their versatility. They allow us to calculate multiple solutions with multiple unknowns, with a simple program structure and its relative ease of implementation [45]. MC simulations are essentially based on non-deterministic models that assign random numbers to trajectories associated with the atoms’ displacements [46]. The Metropolis Monte Carlo (MMC) has become very popular over the years because its use is not restricted only to states of equilibrium but can be extended to calculating dynamic properties [47]. This approach searches for an equilibrium state of the system within probable states generated by a Boltzmann distribution [46]. A second technique with high importance corresponds to the molecular dynamic simulations, which allows determining the equilibrium and transport properties by finding the atoms’ displacement through a numerical solution of Newton’s equations of motion [34]. Some of the most used algorithms in MD correspond to the Verlet, velocity Verlet, and Leapfrog algorithms, which satisfy the symplectic condition [48].

Currently available software packages for MD simulations popular include AMBER [49], GROMACS [50], CHARMM [51], NAMD [52], LAMMPS [53], and DL-POLY [54]. The first four software packages are principally developed for biochemical macromolecules such as proteins, lipids, and nucleic acids. Simultaneously, LAMMPS is focused on materials modeling, and DL-POLY is a general-purpose simulation package [54,55]. The difference between them mainly lies in their performance, capacity, data processing, and adaptability to new hardware. For instance, coupling to GPUs of exceedingly high performance should be easily achievable to shorten simulation times significantly [56].

**Figure 2:**
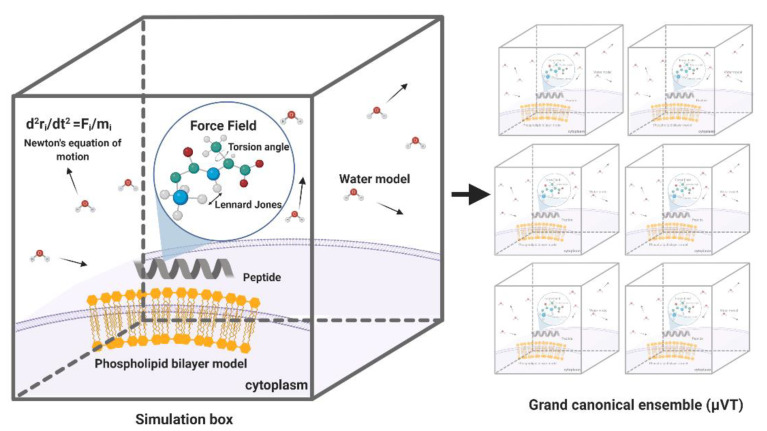
Representation of a traditional membrane-protein system used in molecular dynamics. The interaction among the components is modeled through force fields that account for variations in key parameters and impose restrictions on the accessible states (Created with BioRender).

MD simulations have demonstrated exceedingly high performance in finding information at the atomic level in silico that would be very difficult to obtain experimentally [57]. In the context of our work, this is the case of peptide–lipid bilayer interactions. Therefore, the collected information is valuable to investigate different aspects of such interactions, including the mechanism of action and the toxicity of peptides with antimicrobial and other membrane activities [58]. Moreover, it is possible to conduct experiments in different lipid membrane models, such as bacterial, mammalian, and even carcinogenic [59]. Additionally, diseases involving dependence on the composition of the bilayer, such as cancer, Alzheimer’s, and cardiovascular diseases, can be explored mechanistically in silico to guide the experimental development of novel therapeutic approaches [60,61,62]. An example of a classical representation of a peptide–lipid bilayer system in MD is given in Figure 2.

The last stage of our proposed framework is dedicated to screening potential candidates experimentally via microfluidics platforms. This microsystem family has been comprehensively explored to screen different bioactive compounds, including DNA, proteins, enzymes, receptors, and peptides [63]. The development of platforms for single cells screening, to produce biofuels and drug screening resistance assays [64,65]; biomarkers, involved in the reliable prediction of diseases [66]; screening of bacteria with high production of lactic acid such as *Bacillus coagulans* [67] and library screening for enzyme engineering applications [68,69], are proof of the versatility of this mechanism, showing promising results in the field of biotechnology. In all cases, this approach has been considered advantageous, mainly due to the ability to perform thousands of reactions at the nanoliter to femtoliter scale, replacing robotic automation using small volume samples, reducing unit costs of experimentation, and increasing throughput [70,71,72]. Additionally, microfluidics offers a dynamic integration with different components, allowing the interaction between several variables within a single platform, providing the tools to increase the assays’ precision, accurate determination, and control of experimental conditions. Finally, the ability to handle features in the range of a single cell proportion results in scaled down readouts and a single cell resolution sensitivity [65,70,71,73]. Remarkably, in peptides, microfluidics has reduced reagents utilization and sample consumption, provided shorter times, and fully automatized the process [74]. The implemented microfluidics screening techniques for the case of antimicrobial peptides include three main strategies, namely, droplet-based, membrane-based, and combinatorial microarrays, which are explained in more detail below.

This review aims to critically analyze the latest developments in each of the three main cornerstones of the proposed frameworks, namely artificial intelligence for searching for new candidates, MD simulations to investigate interactions with lipid bilayers, microfluidics to conduct screening of possible candidates in a high throughput manner. We expect the proposed approach to accelerate antimicrobial peptide discovery in a reliable but cost-effective manner to impact the emerging antibiotic resistance issue positively. This is critical before the situation reaches a point-of-no-return where the healthcare system stands hands down against massive uncontrolled infections on a global scale.

## 2. Antimicrobial Peptides

Antimicrobial peptides (AMPs) represent essential components of the higher organisms’ innate immunity; however, they are produced by all lifeforms [75]. AMPs have been isolated from microorganisms, fungi, insects, and other invertebrates, plants, amphibians, birds, fish, and mammals, including humans. These peptides are produced either by ribosomal translation of mRNA or by nonribosomal peptide synthesis, mainly identified in bacteria [75]. AMPs are short sequences (12 to 100 amino acids) that generally exhibit broad-spectrum activity and cationic behavior with a net charge ranging from +2 to +9. Additionally, they are usually amphipathic and, in most cases, present hydrophobicity levels greater than 30% [76]. Lysine, arginine, tryptophan, and cysteine residues are highly conserved throughout their structure. Lysine and arginine have been thought responsible for enabling electrostatic interactions between the peptide and negatively charged membranes.

Additionally, given tryptophans’ unique sidechain containing an indole ring that holds hydrogen-bonding potential, they show strong membrane-disruptive activities by interacting with a membrane’s interface capable of anchoring the peptide to the surface of the bilayer. Regarding cysteine, the disulfide bonds formed are strongly hydrophobic and play an essential role in the peptides’ overall structure and increasing stability towards proteolytic degradation [77]. Given the wide range of antimicrobial activity and varied action mechanisms, AMPs are currently under study as alternative biomolecules to treat infections in scenarios involving resistant microorganisms. Several antimicrobial peptides have been reported in various databases such as The Collection of Anti-Microbial Peptides CAMPR3 (8164 entries) [78], Database of Antimicrobial Activity and Structure of Peptides DBAASP v3.0 (16180 entries) [79] and The Data Repository of Antimicrobial Peptides DRAMP v2.0 (19899 entries) [80]. These peptides can be categorized by their origin, either synthetic or natural, by taxonomy and by activity. According to the DRAMP database, activity classification is divided into four principal classes, antibacterial (7856), antiviral (2015), antifungal (3371) and antiparasitic (148), but also into Anti-Gram+ (2568), Anti-Gram- (2397), anticancer (293), antitumor (156), insecticidal (246) and antiprotozoal (17).

### 2.1. Antibacterial

AMPs with antibacterial activity are the most studied. Antibacterial peptides can be classified into non-ribosomal synthetic peptides and natural or synthetic ribosomal peptides [81]. The first group is mainly produced by bacteria, while the last is produced by all animals and bacteria [82]. Virtually all antibacterial peptides have less than 100 amino acid residues, mainly in the range of three to 50 [83]. The antibacterial peptides structure has four styles, including α helices, β-sheet, extended and looped shapes. The β sheet and the α helix are more abundant in nature [84]. Most of them are cationic with hydrophilic and hydrophobic domains, allowing them to target bacterial cell membranes and cause the lipid bilayer structure’s breakdown. Furthermore, AMPs can kill bacteria by inhibiting some important cell pathways, such as DNA replication and protein synthesis [85].

Many researchers believe that the ability of AMPS to bind to bacterial membranes plays a vital role in their development [86,87]. Some mechanisms for attaching AMP to bacterial membranes include the cane, the toroidal pore wormhole, the carpet pattern, and detergent [76]. The main obstacle in using antibacterial peptides is their ability to lyse eukaryotic cells, especially red blood cells. For their application, they must have low hemolytic activity and high antimicrobial activity [88].

### 2.2. Antivirals

Antiviral peptides are biochemically characterized by being cationic and amphipathic, with net positive charges to effectively work as antimicrobials. Different reports reveal that hydrophobicity seems to be a fundamental property to assure significant activity against enveloped viruses [89]. Antiviral peptides are classified according to their mechanism of action [90]. This includes blocking viral receptors, inhibiting adsorption by antimicrobial binding peptides to viral proteins, interaction with co-receptors such as CXCR4, inhibition of cell fusion by interfering with the protein’s ATPase activity, inhibition of gene expression, inhibition of peptide elongation, and activation of immunomodulatory pathways [18,91,92].

### 2.3. Antifungal

Most antifungal peptides (AFPs) exhibit rapid and potent membrane activity and show a low likelihood of inducing de novo resistance given their wide range of inhibitory mechanisms. As for the other AMPs, AFPs are produced by all living organisms. When generated by unicellular organisms, they are small with a structure containing non-protein amino acids and a fatty acyl moiety. Simultaneously, the AFPs produced by multicellular organisms are more extensive, with the majority having either linear α-helical or cystine-stabilized defensin-like structures. AFPs can be divided structurally into linear peptides, β-sheet peptides, peptides with a mixture of α-helices and β-sheets, and peptides rich in amino acids specific moieties such as modified cyclic peptides, depsipeptides, and lipopeptides [93]. Alternatively, AFPs can also be classified by their action mechanism as membrane-disrupting lytic peptides, which are usually amphipathic and abundant in nature. Cell wall synthesis or bio-synthesis obstructive AFPs are safe and effective for immune-compromised patients [94]. AFPs have also been incorporated into food formulations for preservation purposes [95].

### 2.4. Antiparasitic

Antiparasitic peptides (APPs) are by far the least studied ones. For this reason, there is no recollection of their structural similarities with the other families of AMPs. However, many peptides such as defensins, scorpines, decoralins, drosomycins, cecropins, and Buforin II have been reported as antiparasitic [96,97,98]. For a review on APPs, we encourage the reader to consult [98]. In general, the APP’s action mechanism is associated with selective parasite’s membrane disruption, which usually takes place within the host cell where the parasite is often hidden. Once APPs bind to the host’s membrane, the peptide can transfer to the parasite membrane and exert a lytic activity. Such transferring ability is attributed to the parasite infection’s permeability pathways into the host cells [96].

## 3. Peptide Library Design

The discovery of new AMPs involves preparing peptide libraries, consisting of a large collection of varied sequences to determine critical fragments (motifs) required for specific biological functions. Consequently, they can be used to enable an ample number of applications in proteomics, structure–function relationship studies, vaccine development, epitope mapping, or cancer therapy [99]. Additionally, they offer the possibility to include sequences of modified peptides and peptides containing unnatural or D-shaped amino acids [100,101]. In principle, library-based peptide discovery adheres to the following paradigm: (1) creation of a pooled peptide library, (2) screening of the library against the target molecule and isolation of hits, and (3) hit identification [102]. Peptide libraries can be designed through both rational and non-rational approaches.

### 3.1. Rational

Rational screening aims at creating or discovering peptides by searching for specific physicochemical and biological characteristics or functionalities. This goal can be accomplished either computationally or experimentally. In computational approaches, the libraries can be created with modified and unmodified sequences [102,103]. Decades of work have led to chemoinformatic models that predict molecular properties; however, their accuracy has been insufficient to significantly improve the already established design/discovery process [104]. Nevertheless, given the advances in molecular representations through neural networks (NNs), deep learning techniques are emerging as an alternative to traditional library screening and property prediction [105]. Perhaps the most important advance of NNs concerning chemoinformatics lies in the type of molecular representations they learn. Traditional methods receive manually designed representations as the input, such as different fingerprints that recognize functional groups present in molecules or similarities [104]. In contrast, in deep learning, neural networks are trained to learn an optimal representation from the data for each task by jointly extracting and analyzing many features that an expert may disregard while manually performing the task [33].

#### Deep Learning

Deep learning modeling of peptides is akin to the problem of natural language understanding, as in both cases, the most important information lies in the sequential order of their elements [106,107]. This observation has allowed the application of techniques that were originally designed for language analysis, such as recurrent neural networks (RNNs), to the task of AMP discovery. Within RNNs, long-short term memory networks (LSTMs) and gated recurrent units (GRUs) are the most popular algorithms for peptide activity prediction [108,109,110,111,112]. They both enable analyzing the atoms’ sequence due to the information flow between the current amino acid and the ones analyzed before it. For adequate deep learning experimentation, there are four important aspects to consider: (i) databases, (ii) architectures (the type of neural networks), (iii) molecular-input representation, and (iv) metrics used to evaluate the performance of the models [33]. Regarding overall peptide analysis—not only for AMP discovery—each of these aspects is reviewed below.

Databases. These are fundamental in the design of deep learning algorithms. Based on their data quality, the neural networks can learn useful and generalizable feature representations for specific problems [113]. Consequently, data curation is critical for these techniques to work properly. Many available databases are not standardized or easy to download, thereby leading to an unwanted scenario where each project/problem practically needs to design its dataset [111,114]. Under such circumstances, the comparison between methods and their performance is unfeasible, which has led some researchers to design minimal datasets. In this case, and due to the network capacity, the algorithm can memorize the data, thereby giving a high performance that will not be generalizable to other datasets [33]. A more principled approach is to have the data extracted from larger datasets or to conduct experiments in multiple empirical frameworks. Examples of such databases include the former Antimicrobial Peptide Database (APD), the Collection of Anti-Microbial Peptides (CAMPs), the Anuran defense peptides (DADP), and the CAMEL database [107,108,115,116].

Molecular Input. Molecular input representations can be divided into two groups: (i) based on the peptide sequences and (ii) based on chemical properties. Amino-acid peptide sequences are generally encoded into a one-hot vector of size 20xM. Each row represents the presence (1) or absence (0) of each amino acid on the *Mi* position of the peptide [116,117]. Another common representation is a 1D vector of size 1xM, where each amino acid is encoded by a number N ∈ {1, 2, …, 19, 20} for each essential amino acid [108]. Finally, a representation known as wodr2vec embedding has recently gained increasing popularity. This approach was initially proposed for language processing and consists of representing words from a vocabulary as vectors, whose distance is based on shared similarities [118]. For example, words such as Paris, Madrid, and Rome will map to points closer to each other than Paris, dog, and pizza, because, in the first case, they have something in common: they are all capital cities of a country. In order to extrapolate this representation to peptides, Hamid, and colleagues proposed to establish trigrams of the peptides on three reading frames as words [119].

Regarding chemical properties, representations might significantly vary depending on the ultimate biological task. For instance, in the case of anticancer prediction, common representations include amino acid composition (AAC), dipeptide composition (DPC), composition-transition-distribution (CTD), quasi-sequence-order (QSO), amino acid index (AAIF), binary profile (NC5), and conjoint triad (CTF) [114]. For protein-peptide binding sites prediction, popular choices are half-sphere exposure (HSE), secondary structure (SS), accessible surface area (ASA), local backbone angles, position-specific scoring matrix (PSSM), and physicochemical properties [120]. Specifically, for AMP discovery, peptides have been represented by their topological pharmacophore features, converting a given residue sequence to a 147-dimensional descriptor that encodes the standardized cross-correlated pharmacophore features of the amino acid residues [121]. In this case, the available databases include amino acid composition (AAC), composition-transition-distribution (CTD), general PseAAC (PseAAC-General), and pseudo K-tuple reduced amino acids composition (PseKRAAC) [112].

**Figure 3:**
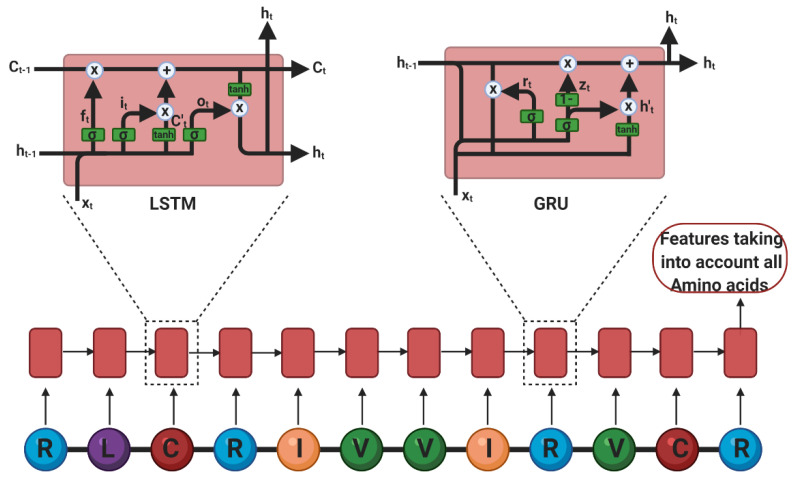
Recurrent neural networks used for peptide property prediction. A representation of each of the amino acids is input to one unit of the recurrent network. The last unit’s output contains information considering the output of the previous ones, therefore considering the whole sequence. On the left a long-short term memory networks (LSTM) unit representation and on the right a gated recurrent units (GRU) unit representation. (Created with BioRender).

Architectures. The most common architectures for peptide analysis are RNNs, mainly LSTMs, in unidirectional and bidirectional configurations. The LSTMs consist of multiple sequential cells with three gates and a cell state (i.e., a memory) to control the flow of information (Figure 3) [122]: (i) Input Gate: Decides what to keep about the input and the previous information. (ii) Forget Gate: Decides what to forget about the information mentioned above and what to forget about the new input. (iii) Output Gate: Decides what to allow the next cell to see.

Equations (1)–(3) models these gates, respectively:

(1) $$i_{t}=\sigma \left(W_{i}\left[h_{t-1},x_{t}\right]+b_{i}\right)$$

(2) $$f_{t}=\sigma \left(W_{f}\left[h_{t-1},x_{t}\right]+b_{f}\right)$$

(3) $$o_{t}=\sigma \left(W_{o}\left[h_{t-1},x_{t}\right]+b_{o}\right)$$

Where *W* represents a weighting matrix for each gate, and *h*, the cell state. The cell state and the output of the cell are given by Equations (4)–(6):

(4) $$c_{t}=f_{t}*c_{t-1}+i_{t}*{c^{\prime }}_{t}$$

(5) $${c^{\prime }}_{t}=\tanh\left(W_{c}\left[h_{t-1},x_{t}\right]+b_{c}\right)$$

(6) $$h_{t}=o_{t}*\tanh\left(c_{t}\right)$$

where W represents a weighting matrix for each gate, c represents a cell state, and h the output of the model [123]. According to this set of equations, the RNN can keep short- and long-term memory about the sequence of amino acids that have been processed, thereby making robust decisions based on the peptide’s full length and primary structure. LSTMs have been successfully applied to activity prediction of multiple peptides in different scenarios such as anticancer [109,124] and subcellular localization targets [111]. Specifically, for AMPs, Veltri and colleagues proposed an NN with three main phases. First, in an embedding phase, peptide sequences were converted to vectors, where each amino acid was assigned to a number. This was followed by an embedding layer that generates three-number vector representations. Second, those representations undergo a feature extractor phase composed of a convolutional layer. Furthermore, the features were analyzed by an LTSM of 100 cells. With this method, the authors were able to outperform the previous state-of-the-art algorithm [108]. More recently, Li and colleagues designed an attentive deep learning model for the discovery of new AMPs. The model, named AMPlify, discovered four novel AMPs that were active against multiple species of bacteria, including a multi-drug resistant isolate of carbapenemase-producing *Escherichia coli*. The AMPlify architecture includes three main components: A bidirectional LSTM, a multi-head scaled dot-product attention (MHSDPA) layer, and context attention (CA) layer. Like any other LSTM, this component encodes positional information for each residue from both forward and backward directions. The MHSDPA layer searches for relations between different residues in various representation subspaces. Finally, the CA layer gathers information from the MHSDPA layer by weighted averaging the encoded vectors of different representation spaces into a single summary vector that provides comprehensive spatial and contextual information. The attention layers in AMPlify play a central in the model’s performance and were considered responsible for outperforming the model put forward previously by Verti et al. [116]. LSTMs have also demonstrated the ability to predict LC-MS/MS1 behaviors of peptides based on a one-hot encoding of their linear sequence [110,117].

Furthermore, gated recurrent units have been implemented to predict antimicrobial activity as they also enable a temporal analysis of the information flow, while needing fewer parameters than LSTMs. Their gates are redefined as follows [125]: (i) Reset Gate: Decides what of the past information is forgotten based on the previous cell state. (ii) Current Memory Content: Uses the reset gate to store relevant information from the previous cells. (iii) Update Gate: Decides what of the past information must go on to the next cell. (iv) Final Current Memory at that time step: Holds the information from the current cell.

In this case, the gates are modeled by Equations (7)–(10), respectively:

(7) $$r_{t}=\sigma (W_{r}x_{t}+U_{r}h_{t-1}+b_{r})$$

(8) $${h^{\prime }}_{t}=\tanh(W_{h}x_{t}+U_{h}(r_{t}h_{t-1})+b_{h}$$

(9) $$z_{t}=\sigma (W_{z}x_{t}+U_{z}h_{t-1}+b_{z})$$

(10) $$h_{t}=z_{t}h_{t-1}+(1-z_{t}){h^{\prime }}_{t}$$

The flow of information for the reset, update, and final state gates are described by Equations (7), (8) and (10), respectively. Additionally, W and U represent weighting matrices and h, the cell state [125]. Hamid and colleagues used a two-layer bidirectional GRU cells to classify peptides between bacteriocins ad non-bacteriocins. Their algorithm takes as input a word2vec embedding to construct the words from trigrams (three consecutively amino acids of a sequence) using three reading frames. Despite a lower performance than that of the state-of-the-art, the authors highlight the superiority of their approach when studying problems where discovery is challenging by sequence similarity analysis with nonhomologous peptides [119].

We have also explored GRU cells to predict organic molecules’ binding to cellular receptors based on a 1D linear representation known as simplified molecular-input line-entry system (SMILES). Our model, PharmaNet, that solely relies on the organic molecules’ information, was able to outperform the state-of-the-art method, which relied on 3D neural networks and cellular receptors [126]. We identified the candidate CHEMBL2007613 (5-([5-Amino-4H-1,2,4-triazole-3-yl]amino)sulfonyl-2-chloro-4-mercaptophenyl acetate) within the ChEMBL database as a potential antiviral treatment due to activity towards the farnesyl pyrophosphate synthase (FPPS). Moreover, CHEMBL2007613 has been reported to upregulate the PCDH17 gene expression, which has also been related to viral infections [127]. PhamaNet highlights the importance of deconstructing molecules into sequences for analysis, thereby enabling information flow between previous and subsequent atoms.

Finally, an emerging architecture for different types of molecules is graph convolutional neural networks, which provides a global understanding of molecules’ structure by a message-passing algorithm that enables information flow between all the atoms and their bonds. With this approach, Stokes and colleagues were able to identify a new antibacterial molecule that is structurally different from known antibiotics and originally intended for diabetes treatment. The new antibiotic shows activity against pan-resistant bacteria, demonstrating the suitability of deep learning techniques to identify functional details that are not evident to human perception [104].

Metrics. Peptide activity prediction is usually evaluated by sensitivity, specificity, accuracy (ACC), area under the receiver operating characteristic curve (ROC-AUC), precision, recall, F1, and/or Matthews correlation coefficient (MCC). Within those metrics, ROC-AUC, ACC, and F1 can lead to overoptimistic results, especially on highly imbalanced datasets. This phenomenon can be explained by their consideration of predictions for true negatives that are not of interest to accomplish the task [128]. Given that these computational approaches’ final objective is to reduce experimental time, it is important to develop and optimize the methods with robust, trustworthy, and stringent metrics. Otherwise, many predicted candidates may not necessarily be active against the targets of interest. To address this issue, we proposed the use of the area under the normalized average precision (NAP) curve as a metric that is stricter and more reliable than conventional precision–recall curves [126].

Deep learning methods have shown some advantages over other machine learning algorithms. Rather than relying on hand-crafted features of the peptides, deep models learn their own features, however, if extra properties are available, they can be input as metadata to enhance the performance even further. Even though state-of-the-art methods have a good performance, their high reliance on hand-crafted features prevents the discovered AMPs to be outside the data distribution of known AMPs. As shown by Stokes and colleagues, the deep learning method can find antibiotics from different data distributions, which would open the search space considerably [104]. Furthermore, traditional machine learning approaches exhibit strong length dependence, assigning very high scores for sequences over 100 amino acids regardless of whether they were AMPs or not [129]. Given that deep learning methods used for peptides discovery were initially developed for natural language processing there is no limitation in the length of analyzed sequences [29,30,31,32]. Additionally, to perform not only binary (i.e., AMP or not AMPs) but a more specialized classification, some state-of-the-art techniques rely on the combination of multiple binary methods. In contrast, deep learning neural networks can learn multiclass classification with a single method [130].

The shallower layers of both RNNs and transformers learn local characteristics of the peptides structure while the deeper ones focus on more global and abstract information related to their functional characteristics [33]. Accordingly, a model trained for property prediction for one specific bioactive peptide should enable transfer learning towards another bioactivity of interest by retraining the deeper and the final layers of the networks. This is particularly interesting for cases where the training data are limited as is the case for antiparasitic and antiviral peptides [131].

### 3.2. Non-Rational

Small-molecule libraries have been widely implemented for drug discovery as a robust route to identify biologically active molecules. Traditionally, small molecule library design is based on a known target structure or known ligands [132]. Peptide-based drugs emerge as safer and more specific alternatives to small molecules and have become a new paradigm in medicinal chemistry [133]. This has attracted significant attention to the development of combinatorial approaches to identify new peptide therapeutics [19]. There have been two different approaches to the construction of random peptide libraries. According to one approach, peptides have been chemically synthesized in vitro through several formats, including phage, *E. coli*, and ribosomal display. For the most part, these synthetic systems have been directed to generating arrays of short length peptides—generally between six to eight amino acids [101]. According to a second approach, peptides have been expressed in vivo via recombinant DNA techniques, as either soluble fusion proteins or viral capsid fusion proteins. In any of these methods, the generated peptide libraries have been suggested to identify peptides exhibiting binding affinity for a chosen ligand [101].

**Figure 4:**
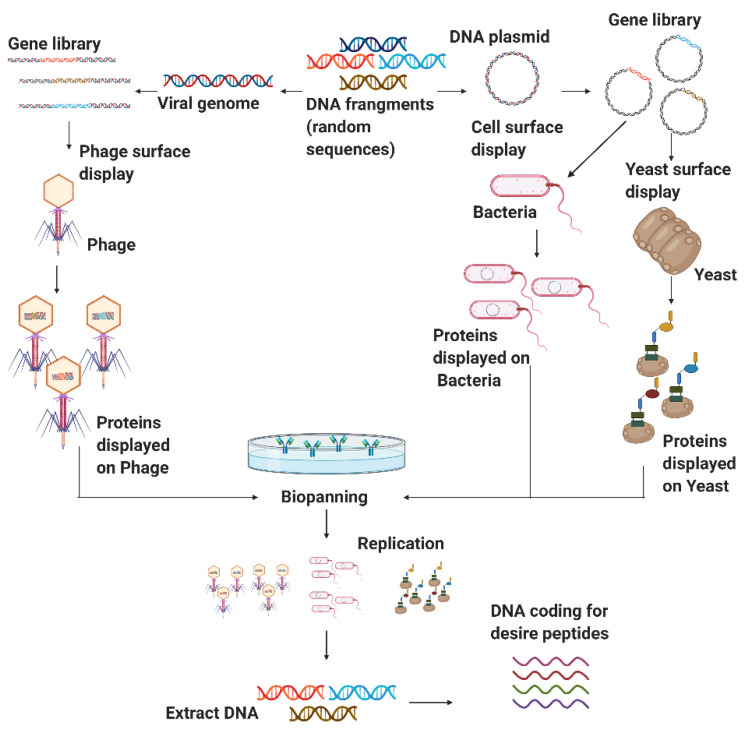
Scheme of the different microbial cell surface display methods. The phage display method is shown on the left, while the bacterial display process is presented in the center, and the yeast display is presented on the right. In all cases, through molecular biology tools it is possible to express the protein fragments of interest quite robustly for further biomolecular interaction analysis and screening (Created with BioRender).

Some random peptides library applications include sequence optimization, enhance antibody epitopes, improve T-cell epitopes, and target identification drug discovery activity [134]. In this context, the microbial surface display is the primary focus for obtaining various well-established random peptide sequences to discover new therapeutics [19]. Microbial cell surface display technology can redesign cell surfaces with functional proteins and peptides to endow cells with unique features. A cell-surface display system contains three main factors, namely host, carrier, and passenger. The host cells serve as the matrix to bind exogenous fusion proteins and an anchoring motif. The carriers are generally outer membrane proteins appended to the cell surface; whose signal peptides can facilitate passengers’ pass from intracellular to the surface. The passengers target foreign proteins for their display on the cell’s surface [28]. Microbial cell surface display technology involves membrane transport, closely resembling the protein secretion mechanism [135]. Typically, host microorganisms can be divided into phages, other bacteria, and yeast. Figure 4 shows a schematic of the general procedure for the generation of non-rational libraries. The process begins with displaying DNA fragments on the surface of the microorganisms. A screening process is then carried to recover the DNA fragments of interest, followed by duplication through replication [136]. Each of the involved processes will be explained in detail in the following sections.

#### 3.2.1. Phage Display

Phage display is a molecular biology technique in which phage DNA is genetically modified to express the peptide of interest on the phage surface. Alternatively, it is possible to express the desired protein fused to one of the phage coat proteins. This strategy is fundamentally different from other bacterial expression systems in that the displayed peptides or proteins and the DNA encoding them are physically linked [137].

The phage display system has many remarkable advantages over other expression systems, such as high throughput biopanning, screening of mimic epitopes, and a simple preparation process [138]. This technology has had a far-reaching influence on protein molecule mutual recognition, vaccine development, and tumor treatments [138]. The power of phage display comes from two distinctive features: (i) the establishment of a physical connection between the phenotype (the displayed peptide) and the genotype (the DNA sequence encoding the displayed peptide) within the same viral particle; and (ii) the production of large and diversified libraries of peptides displayed on the surface of phage particles [139].

The phage display technology has been developed for different bacteriophage systems such as λ, T4, and T7 and the filamentous M13 bacteriophage. Each of these phage systems has its benefits and drawbacks; however, bacteriophages’ (BPs) most attractive characteristic is their specificity of action, i.e., their ability to kill only the recognizable pathogens [140]. In particular, the phage T4 has contributed to many breakthroughs in the fields of genetics and biochemistry. Moreover, recent studies showed that the phage T4 is highly immunogenic and can be exploited to develop potential vaccine candidates [141]. Due to this, they have a very narrow spectrum of activity, thereby avoiding some of the most critical issues of antibiotic administration, which include the influence on the entire microbiome with the elimination of potentially beneficial bacteria, the overgrowth of secondary pathogens, and the emergence of resistant bacteria [142]. The most popular phages for display construction are the filamentous bacteriophages and specifically the M13. This type of phage infects Gram-negative bacteria such as Escherichia, Salmonella, Pseudomonas, Xanthomonas, Vibrio, Thermus, and Neisseria. Moreover, it has a high capacity for replication, accommodate large foreign DNA, and can be genetically modified to expose random small peptides on their surface fused with either the minor coat protein pIII (five copies/phage) or major coat protein pVIII (2800 copies/phage) [139].

However, BPs and their products are non-self-antigens. For this reason, they can be recognized by the immune system and induce responses that reduce their benefits. Additional shortcomings of BPs include the absence of specific activity for a given bacterial strain, difficulty in the production of genomes without integrase genes, and sensitivity to antibiotic-resistant genes, genes for phage-encoded toxins, or genes for other bacterial virulence factors [142]. Moreover, major issues have been identified related to their formulation and stabilization in pharmaceutical preparations, reduced activity due to immune system response, nonspecific distribution in organs and tissues, and limited half-life [143]. In this regard, the wild-type M13 phage has shown a half-life of about 4.5 h in mice, which reduces even further (to a few minutes) after various modifications (e.g., glycosylation or succinylation). The reduction in half-life in the bloodstream and the rapid degradation of modified phages appear associated with their interaction with the corresponding receptors and internalization in cells [144].

#### 3.2.2. Bacterial Display

The bacterial surface display system is incapable of expressing complex eukaryotic proteins, which need post-translational modifications to exhibit activity, including glycosylation and disulfide isomerization [135]. The Gram-negative bacterium *E. coli* is the most frequently used host given the maturity of the tools available for its genetic manipulation and high biomass yields. Outer membrane proteins (OMPs), lipoproteins, and autotransporters are popular carriers for Gram-negative bacteria surface display. OMPs are a class of unique integral membrane proteins that can be found anchored in the outer membrane of bacteria with a predominantly β-barrel secondary structure composed of eight to 26 strands. There are large extended loops between the strands on the extracellular side and short loops on the periplasmic side. These characteristics give OMPs high stability in the membrane and the capability of fighting against extremely harsh environments [145].

OMPs derived from Gram-negative bacteria are ideal carrier proteins to present foreign proteins or peptides on the bacterial cell surface. OMPs are highly robust structures for engineering and developing nanopore channels, surface biosensors, and display libraries. These proteins exhibit high structural plasticity, which is evidenced by their high tolerance to mutations. OMPs have, therefore, served as the basis for various surface expression systems, all of which employ chimeric proteins [146].

Although different OMPs possess different sequences and functions, they share similar structures and biological properties [147]. OMPs sequences’ diversity occurs at the N terminal substantially more than C terminal, and the conserved β signal controls their folding and correct assembly. Different OMP carriers, such as *E. coli* OmpA, OmpF, and the outer membrane protein pore E precursor (PhoE), offer other display systems. Outer membrane protein A (OmpA), an important member of the outer membrane proteins (OMPs) in Gram-negative bacteria, is a key virulence factor that mediates bacterial biofilm formation, eukaryotic cell infection, antibiotic resistance, and immunomodulation. OmpA virulence has been thoroughly studied as it plays key roles in regulating the adhesion, aggressiveness, and biofilm formation of the host’s immune response [145]. OmpF is one of the major outer membrane porin proteins in *E. coli* and is responsible for the passive diffusion of small hydrophilic molecules across the outer membrane. Overexpressed OmpF was identified due to a mutation in the promoter, thus offering an ideal carrier protein system independent of its exogenous inducible expression. In general, the external loops’ amino acid sequences of OmpF are less conserved and might tolerate insertions. Based on its structure and genome protospacer-adjacent motif (PAM) analysis, loop eight of OmpF has been selected as the insert locus for peptide fusion. Therefore, genome editing techniques have been successfully implemented to introduce exogenous gene sequences into loop eight between Lys345 and Leu346 [148].

#### 3.2.3. Yeast Surface Display

As a eukaryotic system, *Saccharomyces cerevisiae* has been successfully employed to express and display dozens of complex proteins in the past decades, offering an easy handling procedure, the stable activity of expressed enzymes, and feasibility to construct large protein libraries [135]. The technique was first validated to enhance existing proteins’ affinity but subsequently proved its effectiveness for isolating de novo molecules from naive combinatorial libraries [149]. In addition to tuning the affinity and specificity of multiple proteins and peptides towards a wide range of targets, yeast surface display technology has also been successfully used for epitope mapping to improve recombinant production. Additionally, it has been used to enhance the stability of the molecules of interest and engineer several enzymes [150,151].

The cell surface display systems have been classified into two main systems. One is the N-terminus-free display where target proteins/peptides are produced as fusions with the secretion signal sequence at the N-terminus and the cell wall-anchoring domain at the C-terminus. The other is the C-terminus-free display, where a secretion signal sequence, a cell wall-anchoring domain, and the target proteins/peptides are fused following this order. The effect of the orientation on the display efficiency and the functional properties might vary depending on the target proteins/peptides [152]. Although diverse yeast strains and various cell wall anchors have been used to display a large variety of protein and peptide scaffolds, the most commonly used as anchor is the *S. cerevisiae* α-agglutinin mating complex, which consists of two subunits referred to as Aga1p and Aga2p. As pioneered by Boder and Wittrup, the classical yeast surface display method relies on the N-terminal fusion of a protein of interest to Aga2p [153].

#### 3.2.4. Library Screening

Screening random peptide libraries effectively identify peptides that can bind target molecules and regulate their function [154]. It can be performed in vitro against various cell types, including cultured cell lines, primary cells isolated from animal models or human patients, or processed cells [143]. These different approaches are compatible with screening libraries, such as functional or affinity-based screening and screening in vitro or in vivo [155]. The most common screening method is biopanning [154]. The target protein is physically immobilized, either directly or indirectly, on a solid support, such as magnetic beads [155].

However, various screening/selection methods are at disposal, depending on the peptide library platform. Screening phage libraries against target proteins can be through in vitro affinity selection. The phage population is incubated with the target protein and then subjected to extensive stringent washing to remove weakly binding and unbound phages [154]. In the same way, phage-display screening has also been successfully carried out in vivo. For example, a CX7C phage-display library was injected into mice 48 h after *Staphylococcus aureus*-induced lung infection to select cyclic peptides with affinity for *S. aureus* [156]. Screening the library in vivo is likely to identify hits that target real-life infections [155].

On the other hand, bacterial display technologies have several potential advantages over phage display. Like phage display, many technologies allow screening or selecting peptides that bind a molecule or cell but do not provide a means to directly assess the functionality and antimicrobial relevance of the peptides or their interactions [157]. However, bacterial libraries allow to screen peptides enzymes like transaminase to determine transaminase activity in real-time, the developed system allows the integration of high throughput screening for transaminase activity of extensive collections of microbial isolates and/or enzymes, and the quantification of substrate conversion by the different biocatalysts [158].

The same happens with most of the protein libraries screened that have relied on cytoplasmic or periplasmic expression in bacteria, which implies that both the substrate and the product travel through the cell membrane [159] or that an additional lysis step is needed to perform the enzymatic assay [160,161]. For this reason, droplet-based microfluidics is becoming an increasingly attractive alternative to microtiter plate techniques for enzymatic high-throughput screening [162].

Those are just a few examples of the different screening methods according to each display platform. In general, they can be grouped into three main categories: in silico, in vitro, and in vivo screening. Its use will be depending on the purpose of the library. Each one of the categories will be explained in more detail in the next sections.

Computational Methods or in silico screening. Structure-based virtual screening refers to in silico identifying potential small chemical molecules with a potential affinity towards a binding pocket within known proteins [163]. Molecular dynamics (MD) simulations have been widely used to assess atoms’ behavior, structural stability, and conformational changes at the atomic level. Alternatively, many important paradigms in medicinal chemistry have emerged from the cheminformatics-based analysis of high-throughput screening data. Instead of focusing on individual molecules, this analysis is generalized to chemotypes (substructures, scaffolds, fragments) and leads to more general rules about (in)activity [164].

In vitro screening. Compared with in vivo screening, in vitro selection is simple, rapid, and effective. This approach offers a high-throughput avenue for identifying multiple peptides that bind specifically to single cells independent of whether they are adherent, alive, or fixed [154]. The whole-cell approach’s advantages for peptide screening include retaining their biological functions and activities, proper folding, preserving the three-dimensional structure, appropriate receptor expression level, and association with neighbor proteins [165]. Simultaneously, in vitro biopanning could identify novel cell surface receptors with unknown biological functions, providing information on specific molecular changes [154]. Typically, peptide libraries screening involves incubating the library with a fluorescently labeled soluble target or target-coated magnetic beads for a specific time to allow binding [165]. This is followed by flow cytometry-based systems such as fluorescence-activated cell sorting (FACS) or magnetic separation techniques such as magnetic-activated cell sorting (MACS) [101].

In vivo screening. Peptides identified via in vivo biopanning may prove to be of better clinical significance, given that they are selected in the disease model of choice. Organ-specific peptides could be isolated by performing biopanning and selection in a living animal [154]. The in vivo biopanning selection is similar to that of the in vitro screening with the difference that the peptide library must be intravenously injected into the animal. This is followed by a period when binding occurs of about 1–2 h, after which the animals are perfused to remove unbound peptide-expressing microbial cells. The desired organs will be collected and homogenized for further analysis. Several rounds of biopanning might be needed to identify tissue-specific peptides [154]. One of the major pitfalls in using in vivo screening technology is that the peptides may not necessarily be translated into humans due to the possible differences in peptide binding between species [154].

## 4. Molecular Dynamics (MD)

### 4.1. Configuration of the System

**Figure 5:**
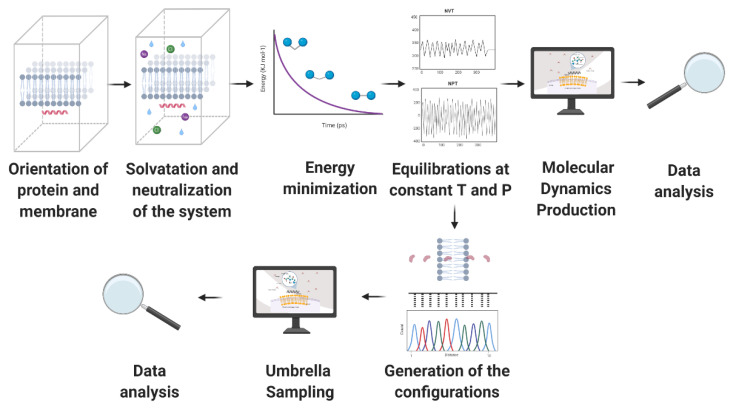
Schematic representation of a classic molecular dynamics simulation (MD) process. Initially, a preparation stage is required in which the system is assembled. Subsequently, the position restraints are turned off to run the MD simulation, and finally, the data of the trajectories are obtained and analyzed (Created with BioRender).

Generally, building a system that aims to simulate phospholipid bilayers consists of selecting a membrane model, the peptide to be evaluated, solvent, and counterions. Periodic boundary conditions are generally enforced to the simulation box to represent a continuum system in the three-dimensional space using a limited number of particles N [166]. The simulation box’s size varies depending on the information intended to be obtained from the MD trajectory. However, orthorhombic shapes have been reported very often with sizes ranging from approximately 4 to 8 nm in width and depth and 7 to 11 nm in height [167,168]. The membrane model can be located either at the center or at one end of the box but is always surrounded by a considerable amount of water to correctly mimicking a biological membrane [167,169]. Figure 5 represents a regular simulation workflow for MD and free energy calculations.

In 2018, Zhao et al. conducted a study in which cathelicidin LL-37, an antimicrobial peptide found in humans, was evaluated for interactions with two different membrane models. The first one consisted of a 1-palmitoyl-2-oleoyl-sn-glycero-3-phosphoglycerol (POPG) model, which aimed to mimic a bacterial membrane due to its negative net charge. The second system comprised 1-palmitoyl-2-oleoyl phosphatidylcholine (POPC) membrane to mimic a mammalian membrane due to its Zwitterionic behavior [170]. A similar methodology was proposed by Wang et al. in 2012, but instead of using only POPG, a mixed model of POPC: POPG was constructed to mimic a bacterial membrane [170]. These two model membranes allowed researchers to evaluate these antimicrobial peptides’ activity against infectious microorganisms and their possible toxicity towards human cells [171,172]. Furthermore, the models have been exploited to gain a much more detailed mechanistic understanding of peptide action.

The first step is to evaluate whether the peptide’s three-dimensional structure has already been determined experimentally. This is verified by exploring different protein databases, such as protein data bank (PDB) [173] or UniProt [174]. These structures are obtained by different methods, including x-ray crystallography, nuclear magnetic resonance (NMR), or cryo-electron microscopy (cryo-EM), among others [37]. However, not all proteins have a reported crystallographic structure because some of these methods are time-consuming and expensive. Additionally, molecules such as transmembrane proteins are quite challenging to crystallize, and some others are not soluble in common solvents [175]. Additional efforts in three-dimensional conformation prediction in silico include platforms such as Iterative Threading ASSEmbly Refinement (I-TASSER), with its de novo predictions [176] and Protein Homology/analogY Recognition Engine V 2.0 (PHYRE2) by homology predictions [177]. This is a critical step, as protein folding broadly defines the corresponding biological function [178].

The peptide is located either inside or parallel to the bilayer to prepare the simulation system, as described by Appelt et al. in 2005 and Zhao [170,179]. Even though some antimicrobial peptides acquire their helical conformation only when they contact the phospholipid bilayer, it is generally assumed that they already have such a structure to reduce the computational cost. However, according to Wang et al., such an initial helicity condition is not required for the lipid–peptide interaction. The folding process will still take place as peptide inserts into the membrane [171].

### 4.2. Molecular Dynamics (MD) Simulations Method

MD simulations are based on the numerical solution of Newton’s equations of motion. A system comprising N molecules can be described by Equation (11).

(11) $$m_{i}r_{i}(t)=F_{i},\;F_{i}=-\frac{\partial }{\left(\partial r_{i}\right)}U(r^{N});\;i=1,\ldots ,N$$

Where $m_{i}$ is the mass of particle $i,r_{i}\left(t\right)$ the position at time $t,F_{i}$ the force acting on particle i, and N the number of molecules. Here, the forces $F_{i}$ that act on the atoms are calculated, which in turn, are originated from the potential energy (U) as a function of their position [36]. The potential energy function (U) is also known as force field. Molecules are defined as a set of atoms that are held together by elastic forces. Multiple force fields are reported in the literature to model systems with different complexity; however, they are generally composed of a series of so-called non-bonded interactions. Force fields (FFs) might also include bonded interactions, which account for local or intramolecular contributions to the total energy. Examples include bonds stretching, angle bending, and dihedral and improper torsions [180]. In the case of Van der Waals interactions, they are usually described by the Lennard Jones pair potential, while for electrostatic charges, the field involves Coulombic interactions. Long-range electrostatic interactions in periodic three-dimensional systems are calculated by the Ewald summation [181] and other related methods such as particle mesh Ewald [182] and Kubic Harmonic expansions [183,184].

A general representation for an equation of a classical force field such as GROMOS, OPLS, or AMBER is presented below in Equation (12) [185].

(12) $$U=\sum_{bonds}\frac{1}{2}k_{b}{\left(r-r_{0}\right)}^{2}+\sum_{angles}\frac{1}{2}k_{a}{\left(\theta -\theta_{0}\right)}^{2}+\sum_{torsions}\frac{1}{2}V_{n}\left[1+\cos(n\phi -\delta )\right]+\sum_{improper}V_{imp}+\sum_{LJ}4\varepsilon_{ij}(\frac{\sigma^{12}{}_{ij}}{r^{12}{}_{ij}}-\frac{\sigma^{6}{}_{ij}}{r^{6}{}_{ij}})+\sum_{elec}\frac{q_{i}q_{j}}{r_{ij}}$$

Each of the classical FFs packages will specify variations in key parameters and the restrictions to be considered. Some other FFs less common will include variations such as the Morse potential replacement by the first term of the equation or changes in the approach to calculate the torsional energy [185]. Once the potential energy function (generally a semi-empirical relationship) has been defined, the equations for the system’s atoms are numerically integrated via different algorithms where initial logical parameters are input to solve the proposed integration scheme [43]. Most of the implemented algorithms have their basis on the Taylor series expansions from the particle position, as it is the case of the Verlet, velocity Verlet, Leapfrog, and the Gear predictor–corrector (GPC) [58,186].

Additionally, the simulations can be carried out mainly at two different resolution levels, dictated by the selected FF. On the one hand, there are atomistic models such as the CHARMM Force Field, which includes all the atoms to represent the system, or semi-atomistic ones such as GROMOS that considers pairs of atoms as single centers for interaction, e.g., CH, CH2, and CH3. On the other hand, the coarse grain (CG) models such as the MARTINI FF in which groups of several heavy atoms represent one interaction center [187]. Simulations run under the MARTINI FF require a significantly lower computational cost, thereby allowing much longer simulation times to be inaccessible under an atomistic representation. This approach’s objective is not oriented towards finding the specific details of involved interactions but to provide a quick, easy-to-use, and versatile route to have such estimates [172]. This can be evidenced by comparing the results presented by Zhao et al. with those of Catte et al. In the first case, simulations under the GROMOS 53a6 FF only reached 4 μs dynamic evolution. In contrast, in the second case, they reached 50 μs with the MARTINI FF [170,172].

The FF’s selection also impacts how the water molecules are modeled to achieve the studied system’s solvation level. When using CG simulations, water is usually represented as a Van der Waals particle, where four water molecules are taken as one coarse-grained bead [188]. In contrast, atomistic approaches allow the modeler to consider different complexity levels and approximations, e.g., incorporate water as flexible or rigid entities. The selection depends on how critical hydration is for the modeled system and the available computational resources [189].

Finally, MD simulations require precise control over temperature and pressure. A typical MD system can be represented by a microcanonical ensemble’s physical characteristics, where the particles N, the volume V, and the energy E remain constant over time (NVE) [190]. Despite the ease of running simulations on the microcanonical ensemble, approaching experimental conditions requires careful consideration of additional parameters. Those are generally constant temperature (canonical or NVT) and constant pressure (isobaric-isothermal or NPT) [191]. Different thermostats can be introduced into the system to control the temperature, such as the Andersen [192], Berendsen [184,193] or Nosé–Hoover [194,195] thermostats [196]. For the pressure control, some common choices are the Rahman–Parrinello method [197], the Berendsen barostat [193], and the Langevin piston [198].

### 4.3. Information Provided by the MD Simulations

MD simulations of lipid bilayers provide insights into the dynamics of involved molecules, e.g., the lateral diffusion of the phospholipid molecules within the fluid membrane, which gives a “qualitative picture” of the molecular mobility [199]. The diffusion can be calculated from the Einstein relation shown in Equation (13).

(13) $$D_{T}=\underset{t\to \infty }{\lim}\frac{1}{4tN_{species}}{\sum_{i=1}^{N_{species}}\left[\vec{r_{i}}(t)-\vec{r_{i}}(0)\right]}^{2}$$

The factor 4 corresponds to the diffusion in two dimensions and the term $\frac{1}{N}{\sum_{i=1}^{N_{species}}\left[\vec{r_{i}}(t)-\vec{r_{i}}(0)\right]}^{2}$ the mean-squared displacement (MSD), which is estimated by adding the displacement contribution from the center of mass (COM) from N species at a time t of the directions. Shahane et al. reported obtaining lipid lateral diffusion coefficients by simulating antimicrobial lipopeptides in the presence of bacterial membranes by calculating the linear adjustment of the obtained mean-square displacement. For instance, this approach allowed determining the lipids’ movement along the XY plane of a bacterial bilayer composed by a 2:1 ratio of phosphatidylethanolamine (POPE) to phosphatidylglycerol (POPG) phospholipids. Their results demonstrated that POPE phospholipids have a higher diffusion coefficient than POPG. Additionally, it was found that there is a strong correlation between the choice of FFs and the simulation conditions with the calculated values for the lateral diffusion coefficients [200].

The simulations also enabled establishing the membrane’s destabilization and penetration into the membrane’s interior through the formed pores. This can be observed by calculating the mass density profiles, which show the mass distribution along the *z*-axis of the membrane [199]. As indicated by Appelt et al., the profiles show where the strongest interactions between the peptide and the system’s main components happen over time, which can be used to determine if the peptides managed to translocate into the membrane or remained excluded at the interface with the water [179]. The water’s density profile is also related to the free energy barrier that the peptide needs to overcome, estimated according to Equation (14).

(14) $$\Delta G\left(z\right)=-kT\ln\rho_{water}\left(z\right)$$

where k is the Boltzmann constant, T the simulation temperature, and $\rho_{water}\left(z\right)$ the water density along the *z*-axis.

**Figure 6:**
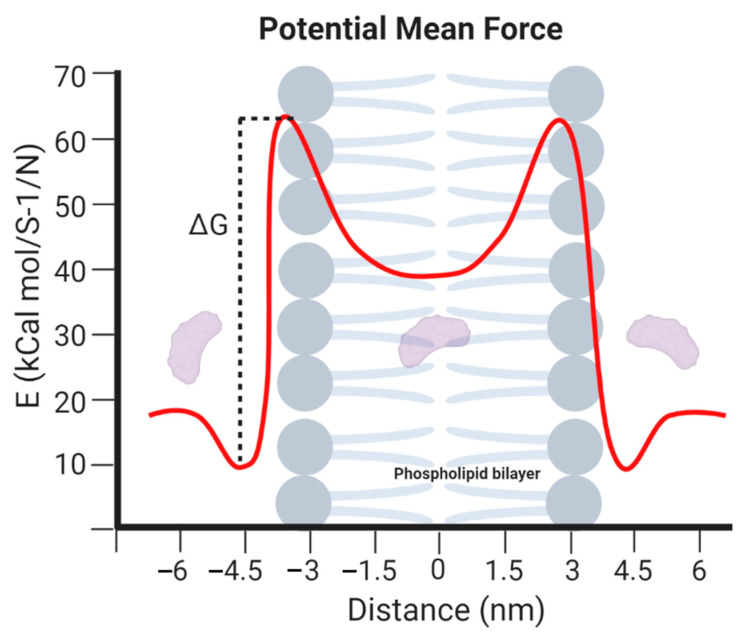
Potential mean force curve calculated from an umbrella sampling simulation across a phospholipid bilayer model where the zero represents the center of mass (COM) of a 6 nm thick membrane (Created with BioRender).

MD simulations are, therefore, useful to quantify the free energy required for any molecule to cross the cell membrane. Depending on the value of this parameter, it is possible to establish whether they are capable of spontaneously translocating the membrane or not. The free energy can be determined by the mean force potential (PMF) curves, calculated based on the distance between the peptide’s COM and the lipid bilayer, as shown below in Figure 6. Such graphical representations can be recovered from an umbrella sampling simulation in which the molecule of interest is constrained by exposing it to an external harmonic potential [201]. By using potentials, recent reports describe the membrane penetration of several antimicrobial peptides such as Indolicidin. Moreover, this route has also been exploited to estimate membrane fusion potential of synthetic peptides E and K and even the permeation of smaller molecules such as bisphenol A [202,203,204].

## 5. Microfluidic Approaches

Microfluidic platforms developed over the past two decades have significantly impacted biomedical research, therapeutics, and diagnostics. The development of such platforms has facilitated the screening processes of peptides with biological activity mainly due to the reduction in reagent consumption, shorter processing times, and the possibility of automation to collect in-line and real-time data [74]. This has led to discovering and testing thousands to millions of new molecules at an unprecedented pace. Moreover, the most recent developments have resulted in compact, traceable, and addressable microsystems to perform thousands of parallel reactions in low volumes of reagents [205]. For the specific case of the research in antibacterial and translocating peptides, the screening has been enabled mainly by three types of schemes: droplet-based, membrane-based, and combinatory microarrays [206,207].

### 5.1. Droplet-Based Screening

This technique has been extensively used due to shorter processing times, lower costs, higher sensitivities, and reproducibility when analyzing hard to detect and low concentration compounds in cell-based assays [208]. In addition to the compatibility with fluorescence-activated sorting (FACS) and the possibility of being incorporated into high-throughput assay systems [209,210]. The principle behind this type of platform is to compartmentalize reactants in picolitre volumes of emulsion droplets instead of the typically employed microliter volumes of most standardized assays [208]. The reactants’ compartmentalization is carried out utilizing an inert carrier fluid such as oil to encapsulate small volumes of the aqueous reagents in droplets. This encapsulation process prevents undesirable interactions between reactants and solid surfaces, decreasing fouling and cross-contamination between samples [208,211]. These droplets have been widely studied to encapsulate cells, viruses, bacteria, and other biomolecules like DNA and peptides [212,213,214,215,216,217,218].

For the study of cell-penetrating peptides, Safa et al. presented a novel microfluidic droplet trapping array platform manufactured in polydimethylsiloxane (PDMS) to perform a single-cell analysis of peptides (e.g., TAT, ARG, RWRWR, and OWRWR) uptake in cancer cells. The cells were incubated with the peptide solutions for 60 min at 37 °C in a CO2 incubator under dark conditions and then injected into the droplet generation microfluidic device’s aqueous inlet to generate the droplet trapping array. The obtained encapsulates were subsequently imaged via fluorescence microscopy [219].

**Figure 7:**
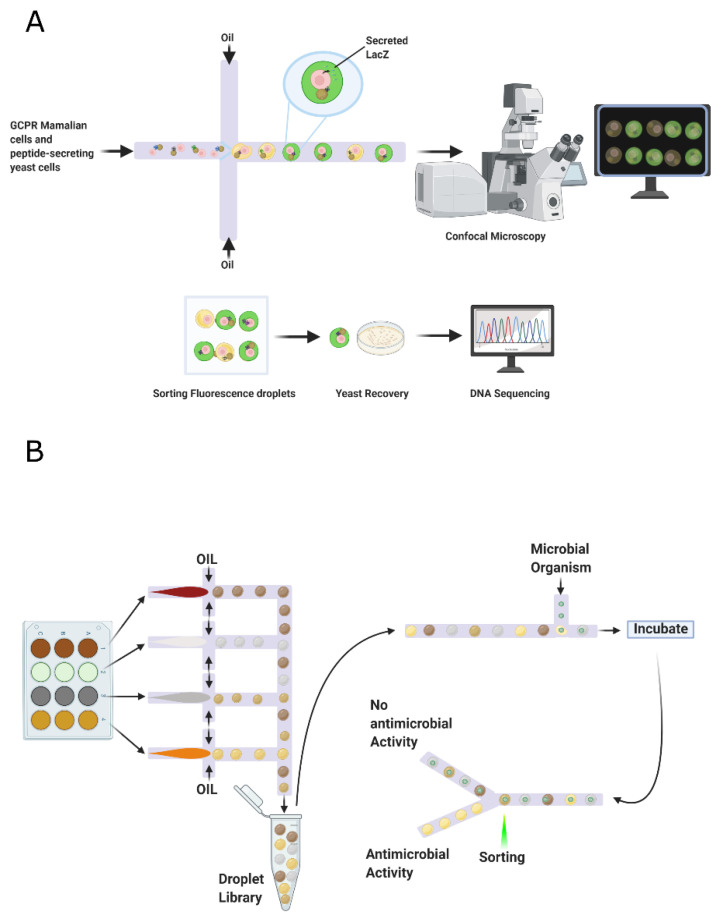
Droplet-based microfluidics screening strategies. (**A**) Schematic of screening performance used by Yaginuma where cells and peptide secreting yeast are encapsulated in a droplet system. Droplets with fluorescence due to the secretion of LacZ are sorted and recovered for yeast culture. Finally, peptides secreted are sequenced to obtain functional candidates. (**B**) Schematic of screening performance used by Guo where a droplet library is generated using a microfluidic approach. The antimicrobial activity analysis is carried out injecting the droplets in a microfluidic platform where single microbial cells are added and incubated. Finally, the droplet-encapsulated compounds are screened and sorted based on the microbial growth arrest (Created with BioRender).

Yaginuma et al. presented a novel droplet-based microfluidic platform for the high-throughput identification of peptide agonists against G-protein coupled receptors (GPCRs) by co-culture of mammalian reporter cells and peptide-secreting yeast cells (see Figure 7A). In this study, the reporter cells and yeast cells that secrete randomized peptide ligands were encapsulated into droplets and co-cultured. When a secreted peptide ligand activates the reporter cell, a droplet emits strong fluorescence by the present reporter proteins (LacZ). When a droplet emits fluorescence, it is isolated such that the entrapped yeast cells can be further cultured. The functional peptide ligands secreted are then finally sequenced [220].

Guo et al. conducted a compound screening aided by droplet libraries. In this approach, the compound’s droplet library is generated by a microfluidic device and pooled together. The obtained droplet library is then screened for antimicrobial activity by injecting the droplets in the microfluidic platform and single microbial cells added to each droplet. The droplet-encapsulated compounds are screened for growth arrest after an incubation stage, which allows selecting antimicrobial candidates [208]. Figure 7B shows the screening platform implemented by Guo et al.

Droplet-based microfluidics has been applied to high-throughput screening (HTS) of enzyme libraries secreted by yeast. This is the case of Sjostrom et al., who introduced a droplet screening method for selecting improved hosts of industrial enzymes. Their system consisted of two main components: a droplet generation circuit and a fluorescent-based sorter circuit. The screening process starts by encapsulating single cells from the whole-genome mutated library and a fluorogenic substrate into microfluidic droplets. This promoted the interaction between each cell’s phenotype and genotype while maintaining encapsulated the secreted enzyme and the fluorescent compound. Yeast clones with an α-amylase production higher than the mother strain can be isolated by looking at the droplet’s fluorescence intensity. The droplets of interest are separated and collected by passing them through a filter that activates an electric field that facilitates the sorting process. This method showed a throughput over 300 times higher than that obtained with a conventional microtiter plate system [221].

Under a similar principle, Beneyton et al. developed an HTS microfluidic platform that exploited the secretion abilities of *Yarrowia lipolytica* and was composed of a drop generator and an integrated screening device. The first step was to encapsulate single yeast cells in 20 pL droplets, then cells were cultured in the same droplets off-chip for 16 h at 28 °C, allowing enzyme secretion. The loaded droplets were injected into the integrated screening device and the fluorogenic substrate for the reaction to occur. The fluorescence emitted was analyzed to sort yeast strains according to their endoxylanase, cellobiohydrolase, and protease activity. The system demonstrated exceptional performance, reliability, and low variability for screening enzyme libraries [167].

Exploring the scope of microfluidics droplet-based screening, Yu et al. studied the high-throughput phenotyping of plant single cells [222]. They developed a platform for characterization and screening of individual plant yellow fluorescent protein (YFP)-expressing protoplasts derived from *Marchantia polymorpha*, which were encapsulated individually via flow-focusing microfluidics in aqueous droplets [223]. Given that light energy absorbed by chlorophyll molecules could be re-emitted as a light signal [224], a fluorescence sensor was integrated into the system to detect chlorophyll or YFP fluorescence activity after laser excitation at 642–682 nm and 488–512 nm, respectively. As fluorescence is emitted, a pulse generator connected to a high-voltage power supply is triggered, resulting in droplet deformation and targeting a small “positive” channel for collection. The empty droplets sent no signal and passed through to a separate compartment. In conclusion, the study demonstrated the feasibility of high-throughput screening for protoplasts as a function of genetic circuit activity or in response to environmental stimuli [225].

### 5.2. Membrane-Based Screening

Membranes are defined as porous barriers that allow the passage of different types of compounds into the intracellular space. Membrane technology has shown several advantages, such as ease of operation, cost-effectiveness, and the possibility of acting as a simplified cell membrane model [207]. Two of these membrane models are the artificial planar lipid bilayers and the liposomes. The former has been widely implemented in the research on membrane proteins, while the latter fulfills the requirements of well-defined lipid composition and easy imaging [226]. Recently, microfluidic technologies have been explored as an alternative for high-precision manufacturing of membrane models to enable high-added-value applications in various industries, e.g., pharma, cosmetics, and food [207]. In liposome synthesis, microfluidics platforms have overcome many limitations of the bulk methods such as batch-to-batch variability, low encapsulation efficiency, and high polydispersity [226,227]. For membrane screening applications, lipid vesicles’ synthesis is commonly carried out by the microfluidic octanol-assisted liposomes assembly method (OLA) [226,228].

**Figure 8:**
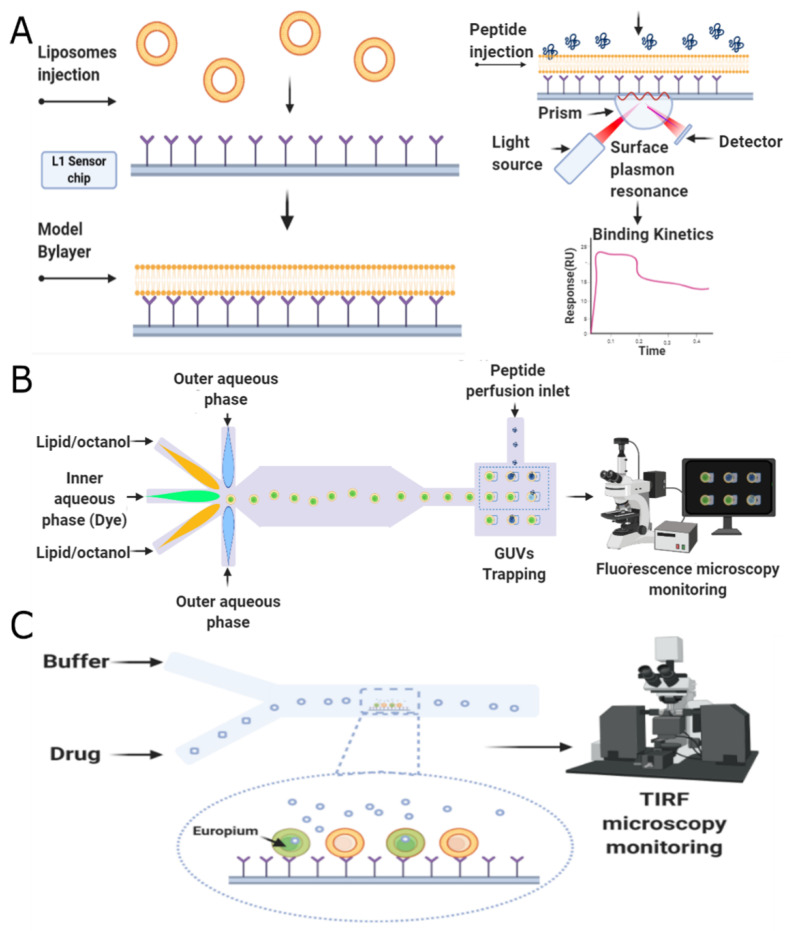
Membrane-based approaches for bioactive compounds screening. (**A**) Surface plasmon resonance (SPR) interaction analysis carried out by Hall et al. and Šakanovič et al. where a lipid planar bilayer is generated in the surface of a sensor chip in which the peptide membrane interactions are measured by surface plasmon resonance [231,232]; (**B**) Membrane-based screening using giant unilamellar vesicles (GUVs) as described by Nahas et al. where GUVs are generated by the octanol-assisted liposomes assembly method (OLA) technique and immobilized in a chamber where the peptides are injected in order to analyze the membranolytic activity by the leak of a dye previously encapsulated into the GUVs lumen. (**C**) Vesicle screening platform used by Kuhn where the GUVs are immobilized onto a glass-bottom and a delivery of the tetracycline is carried out to analyze the drug permeation using a total internal reflection fluorescence (TIRF) microscopy due to the red fluorescence complex generated by Europium-tetracycline binding.(Created with BioRender).

For the artificial planar lipid bilayer, the assembly is carried out across a tiny aperture opened in solid support [229]. Although there have been attempts to reconstitute artificial bilayers in polished micromachined apertures of highly controlled diameters, it is a reasonably skilled process [229]. Nevertheless, Funakoshi et al. reported a simple microfluidic platform for forming a bilayer in the absence of apertures by only controlling the system’s fluidics for the membrane-protein assembly and interaction analysis. They introduced two different configurations for the lipid bilayer formation, which was confirmed by capacitance and ion signals measurements through peptide channels that had been reconstituted into the bilayer [229]. Another example was presented by Zagnoni et al., where an array of lipid bilayer membranes was formed by a microfluidic system for further proteomics applications [230]. One example of the application of planar lipid bilayers in protein research is the research reported by Hall and Aguilar, as presented in Figure 8A. An analysis of the antimicrobial peptide melittin’s membrane interaction was carried out using the surface plasmon resonance (SPR) spectroscopy. The method is based on forming a model bilayer by injecting liposomes in an L1 sensor chip (Biacore-GE Healthcare, Uppsala, Sweden) and the quantitative analysis of membrane interactions measured by the change adsorbed mass at the sensor surface [231].

One example of the application of planar lipid bilayers in protein research is reported by Hall and Aguilar (Figure 8A). An analysis of the antimicrobial peptide melittin interaction with lipid bilayers was carried out aided by surface plasmon resonance (SPR) spectroscopy. The method relied on forming a model bilayer on an L1 sensor chip (Biacore-GE Healthcare, Uppsala, Sweden) after injecting liposomes. The membrane interactions were quantified by measuring the change in adsorbed mass at the sensor surface [231].

A similar study was carried out by Šakanovič et al., in which the interactions of proteins with lipids and lipid membranes were also analyzed via SPR. They used two approaches. In the first one, a hybrid lipid bilayer (i.e., a monolayer of phospholipids supported by a hydrophobic alkane layer) was formed on a HPA sensor chip after a solution of small unilamellar vesicles was injected across the surface of the chip. The second approach followed Hall and Aguilar’s work, where an L1 chip captured intact liposomes on its surface. According to Šakanovič et al., the most crucial advantage of SPR over other biophysical approaches is determining the apparent rate and affinity constants from sensorgrams that can be very useful to study the mechanisms of pore-forming proteins and peptides [232].

Al Nahas et al. presented a novel microfluidic platform for the characterization of membrane-active antimicrobials (see Figure 8B). The platform adapted the OLA method for the high-throughput formation of giant unilamellar vesicles (GUVs), which are immobilized downstream in chambers connected to perfusion inlets through which different solutions of Cecropin B (native antimicrobial peptide) can be injected. This study was carried out to quantify the peptide’s membranolytic activity by measuring the fluorescence of a dye encapsulated in the GUVs as it leaks upon peptide-induced rupture. The results showed a fully integrated microfluidic platform that tests the efficacy of the antimicrobial peptides, either native or designed, that lyse or induce pore-formation in the biomimetic vesicle membranes [228].

Under a similar principle, Kuhn et al. reported a microfluidic vesicle screening platform that determines small molecules’ uptake rates into GUVs. The platform is based on the immobilization of GUVs onto a glass-bottom followed by the delivery of the tetracycline by laminar flow. The image of the drug permeation is achieved by a red fluorescence complex generated due to the tetracycline binding to europium encapsulated inside the vesicles, monitored by a total internal reflection fluorescence (TIRF) microscopy setup [228]. Figure 8C shows a schematic of this screening system. Even though the platform was not designed to screen for peptides activities, its principle can be extrapolated to antimicrobial and translocating peptides. This could be achieved by replacing europium with a fluorochrome to monitor its leakage, as described in the work by Nahas et al. [228].

Finally, Schaich et al. presented an integrated microfluidic platform that generates GUVs by the OLA technique and an optofluidic transport assay. This work studied the transport of norfloxacin and ciprofloxacin through biomimetic liposomal membranes of GUVs aided by ultraviolet video fluorescence microscopy to quantify their uptake and calculate the corresponding permeability coefficients [226]. Even though the study’s main results were not focused on peptides, they reported on an experiment that tested the membrane unilamellarity in the presence of the peptide cecropin B to measure permeabilization and the lysing of the OLA-produced liposomes. These findings strongly suggest that this system can be possibly implemented in the biophysical study of antimicrobial and translocating peptides.

### 5.3. Combinatorial Microarray Screening

Combinatorial chemistry refers to a set of techniques related to a chemical synthesis that allows the preparation of compounds on a large scale in a single procedure that starts from mixture libraries [224]. Research in this area has led to the emergence of microarray technologies, which have provided insights into molecular interactions, drug development, and proteomics [233]. Microarrays can be defined as a library of immobilized compounds such as peptides or proteins displayed on a solid surface to conduct biomolecular interaction studies. This approach has attracted significant attention mainly due to advantages such as spatially addressable studies, highly miniaturized systems, low requirements of analytes, and sophisticated instrumentation. Therefore, microarrays have enabled the study of binding properties, functionality, and kinetics involved in protein–protein interactions [233]. Among a wide variety of technologies based on combinatorial chemistry, the one-bead one-compound (OBOC) method has gained popularity over the years because it allows high throughput synthesis and screening millions of compounds in short periods. OBOC is a spatially separable chemical microarray where only one peptide is displayed on each bead [234]. In this scheme, ligands identified are resynthesized and immobilized on a plate in a microarray format. Multiple probes are employed in multiple replicate sets under different conditions to analyze the respective ligands [233].

**Figure 9:**
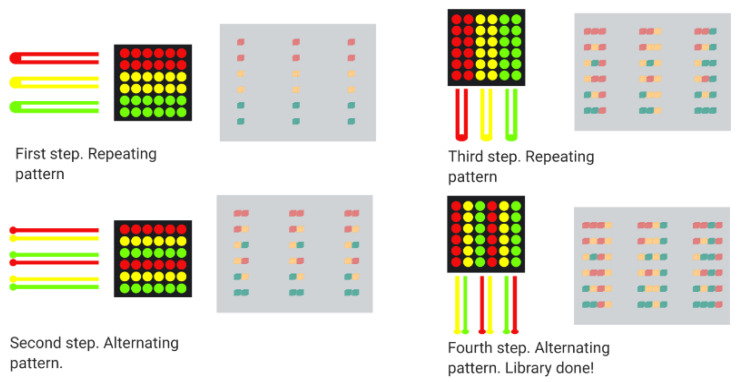
Schematic of combinatory microarray screening performance used by Zhao where possible permutations of the combinatorial peptide library are obtained using different combinatorial flow patterns of amino acids applied to a blank microdisk array. (Created with BioRender).

Combinatorial chemistry has expedited the discovery of modern therapeutics for cancer treatment and diagnostics due to the rapid synthesis of many compounds with particular biological functions or properties [233]. For this reason, Zhao et al. developed a microfluidics-enabled combinatorial peptide library for HTS, which comprises a microdisk array where each component contains a chemical signature and displays a unique numerical barcode that enables a facile identification of the chemical structure. In this study, the synthesis and screening of a random library against α4β1 integrin-presenting cancer cells were performed. The principle is presented in Figure 9. Briefly, different combinatorial flow patterns of amino acids, repeating for the first and third steps and alternating for the second and fourth steps, are applied to a blank microdisk array to couple the substrate’s building blocks. This ensures enough opportunities for the generation of all possible permutations of the library. The chosen peptides are straightforwardly read out from the microdisks’ standardized tag after evacuating the connected cells with a denaturing guanidine hydrochloride buffer [234].

Although OBOC libraries have proven to be useful in identifying novel peptide ligands, the bead hits’ isolation process is performed manually. Consequently, millions of library beads need to be analyzed in a time-consuming and labor-intensive task. By taking this into account, Wang et al. decided to implement an integrated and automated microfluidics screening platform that comprises the high-throughput positive peptide isolation, sorting, and single bead trapping. A mixture of the peptide library beads, biotinylated aminopeptidases N (APN), and the magnetic beads are loaded into the microfluidic chip. In contrast, a magnetic field is applied to trap the positive beads. Then they are separated from the negative ones due to the sheath flow configuration. Therefore, the ligands with the high affinity for the target protein could be isolated employing magnetic separation approaches in a continuous-flow microfluidic process. Finally, the system incorporates in situ MALDI-TOF mass spectrometry to sequencing and identifying noncanonical affinity peptide ligands from OBOC libraries toward the tumor marker APN. This demonstrates a practical and universal strategy for screening peptide probes for different biological systems [235].

Due to the OBOC limitations in terms of high-throughput, recent reports have explored various alternatives, especially from the field of microfluidics [236]. On this matter, Li et al. presented an innovative microfluidic combinatorial synthesis platform called microfluidic print-to-synthesis (MPS) or microfluidic impact printing (MI) [237,238]. This system provides an efficient way to develop an array of specifically designed peptide sequences in an automated and high-throughput manner. The device integrates a multichannel microfluidic cartridge and a pneumatic mechanism, which triggers a three-way electromagnetic switch that directs compressed air to a determined microchannel for droplet printing. They demonstrated the system’s capability by generating functional peptide libraries screened with Jurkat lymphoid malignant T-cells for α4β1 integrin targeting [237]. In this case, the process started by the chemical printing onto the polyethylene glycol (PEG) microdisk array immobilized on a silane-coated substrate. Then, peptide synthesis is carried out by the printing of 9-fluorenylmethoxycarbonyl (Fmoc)-protected amino acids and coupling reagents onto the disc, followed by a washing step and the removal of Fmoc-protecting groups. This process is repeated until all the required peptides of the library are obtained [238].

**Figure 10:**
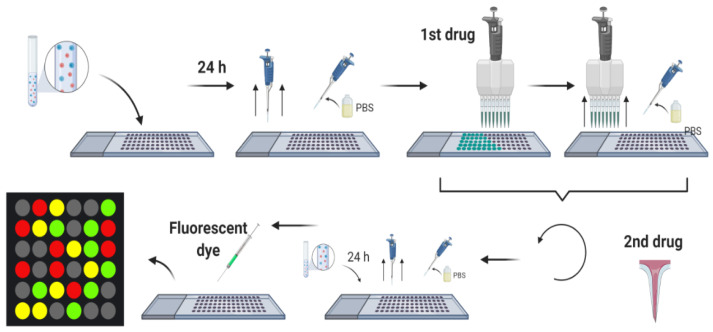
Schematic of combinatory microarray screening performance used by Du et al. where droplets formed in a Nanowell chip by the interaction of cell suspension and oil are exposed for changes in media and schedules dosage-dependent drug assays to perform stimulation–response studies based on the fluorometric analysis in a high-throughput manner. (Created with BioRender).

Finally, the need for large numbers of activity and toxicity tests when performing drug combination therapy represents another field where microfluidics HTS systems could be potentially applied. In this regard, droplet-based screening and the combinatorial method have been reported to play an important role [239,240,241]. Although droplet-based systems have demonstrated their applicability in combinatorial assays, continuous flow configuration fails to provide the flexibility needed to change the flowing droplets’ media. For this reason, Du et al. introduced an integrated microfluidic system based on the sequential operation droplet array technique, which performs the cell culture, changes the media, schedules dosage-dependent drug assays, and studies stimulation responses. The system was tested by screening for A549 non-small lung cancer cells with synergistic combinations of the anticancer drugs flavopiridol, paclitaxel, and 5-fluorouracil. The chosen peptides are straightforwardly read out from the microdisks’ barcode after a detaching cell process from the positive microdisks aided by a guanidine hydrochloride buffer [234]. The first dosage is applied and incubated, followed by media change and stimulation for the second round of drug dosage. This process is repeated several times, corroborating the system’s capability to perform cell-based and schedule-dependent drug combination screening [242]. This combinatorial screening process is shown in Figure 10.

Finally, Table 1 mentions some peptides that have been discover by each of the two cornerstones of the proposed framework. On all the cases, the potential AMP was validated in vitro to corroborate its activity against multiple organisms.

## 6. Concluding Remarks

The indiscriminate use of antibiotics has led to the emergence of antibiotic-resistant microorganism strains that are difficult to treat and cost thousands of lives a year worldwide. Only in the U.S., complications related to such microorganisms led to costs that approached USD 20 billion in 2019 and is expected to increase worldwide to USD 1 trillion by 2050. To complicate matters even further, large pharmaceutical companies have stopped developing new antibiotics due to the considerable investments needed, long payback times, and the high failure rate. These issues have spurred significant research efforts that identified antimicrobial peptides (AMPs) as potential alternatives to address this currently growing healthcare crisis. Despite the attractiveness of AMPs, identifying promising candidates is usually a time-consuming and tedious task because it involves screening large libraries of randomly or rationally designed sequences. Here, we put forward the notion that this significant hurdle can be addressed by a four-stage workflow process that incorporates the interplay of recent advances in four major emerging technologies, namely artificial intelligence, molecular dynamics, surface-display in microorganisms, and microfluidics. The first two can be grouped into in silico strategies while the last two correspond to experimental approaches.

Recurrent neural networks (RNNs) could provide a reliable route for screening large databases of peptide sequences, based on deep learning architectures trained to recognize features in the sequence that might be typically disregarded in importance for the task by humans. The obtained sequences could then be analyzed via molecular dynamics (MD) simulations to search for specific interactions with membranes correlated with the desired biological activity. The candidates with the highest potential will be then expressed on the surface of bacteria or yeasts. They can then be screened for activity using model bilayers produced and manipulated within fully instrumented and smart microfluidic platforms. The present contribution discusses the most recent developments in each of the enabling technologies comprising our methodology. We are confident that the proposed approach will accelerate the discovery of more potent AMPs and provide a robust platform to search for functional sequences to tackle the need for therapeutic approaches in an ample variety of diseases ranging from neurodegenerative disorders to autoimmune conditions.

## Figures and Tables

**Table 1:** Peptides previously designed and evaluated for their antimicrobial capacity under the mentioned methods where MD corresponds to molecular dynamics, DL to Deep Learning and MF to Microfluidics.

Peptide	Amino Acid Sequence	Biological Activity	Method
CAMEL0 [243]	KWKLFKKIGAVLKVL	Antimicrobial	MD
CAMEL17 [243]	KWNLNGNINAVLKVL	Antimicrobial	MD
LL-37 [175]	LLGDFFRKSKEKIGKEFKRIVQRIKDFLRNLVPRTES	Antimicrobial	MD
AcrAP1 [244]	FLFSLIPHAISGLISAFK	Antimicrobial	MD
Chrys-3 [177]	FIGLLISAGKAIHDLIRRRH	Antimicrobial	MD
Pin2 [245]	FWGALAKGALKLIPSLFSSFSKKD	Antimicrobial	MD
Melittin [246]	GIGAVLKVLTTGLPALISWIKRKRQQ	Antimicrobial	MD
Putative Bacteriocin 3 [247]	IKKIGKKAAKKVIVKAIQAI	Antimicrobial	DL
Putative Bacteriocin 4 [247]	KKIGKKAAKKVIVKAIQAIV	Antimicrobial	DL
RaCa-1 [116]	GLLDIIKTTGKDFAVKILDNLKCKLAGGCPP	Antimicrobial	DL
RaCa-2 [116]	FFPIIARLAAKVIPSLVCAVTKKC	Antimicrobial	DL
RaCa-3 [116]	GLWETIKTTGKSIALNLLDKIKCKIAGGCPP	Antimicrobial	DL
RaCa-7 [116]	FFPRVLPLANKFLPTIYCALPKSVGN	Antimicrobial	DL
Cecropin A [248]	KWKLFKKIEKVGQNIRDGIIKAGPAVAVVGQATQIAK-NH2	Translocating/Antimicrobial	MF
Cecropin B [228]	KWKVFKKIEKMGRNIRNGIVKAGPAIAVLGEAKAL-NH2	Antimicrobial	MF
Smp43 [249]	GVWDWIKKTAGKIWNSEPVKALKSQALNAAKNFVAEKIGATPS	Antimicrobial	MF
Cinnamycin [250]	CRQSCSFGPFTFVCDGNTK	Antimicrobial	MF
RWRWR [218]	Ac-RWVRVpGO(FAM)WIRQ-NH2	Traslocating	MF
OWRWR [218]	Ac-OWVRVpGO(FAM)WIRQ-NH2	Traslocating	MF
